# Comparison of three insulin bolus calculators to increase time in range of glycemia in a group of poorly controlled adults Type 1 diabetes in a Brazilian public health service

**DOI:** 10.1186/s13098-022-00903-z

**Published:** 2022-09-13

**Authors:** Vanessa Araujo Montanari, Mônica Andrade Lima Gabbay, Sérgio Atala Dib

**Affiliations:** Endocrinology Division of Universidade Federal de São Paulo-UNIFESP, São Paulo, Brazil

**Keywords:** Insulin bolus calculator, Type 1 diabetes, Mobile applications, Glycemic control

## Abstract

**Background:**

A main factor contributing to insufficient glycemic control, during basal/bolus insulin therapy, is poor self-management bolus. Insulin bolus administration frequency is strongly associated with glycated hemoglobin (A1c) in Type 1 Diabetes (T1D). In the present study, we analyzed the performance of two-bolus calculator’s software that could be accessible to T1D patients from a Public Health Service to improve glycemic time in range (TIR) and A1c.

**Methods:**

This prospective, controlled, randomized, parallel intervention clinical trial was carried out with 111 T1D participants on basal/bolus therapy [multiple daily insulin injections (MDI) or subcutaneous infusion pump (CSII)] with basal A1c ≥ 8.5% for 24 weeks. Patients were divided into 3 groups: 2 interventions: COMBO^®^ (bolus calculator) and GLIC (mobile application) and 1 control (CSII group). Anthropometrics and metabolic variables were assessed on basal, 3 and 6 months of follow-up.

**Results:**

TIR was increased in 9.42% in COMBO group (29 ± 12% to 38.9 ± 12.7%; p < 0.001) in 8.39% in the GLIC® group (28 ± 15% to 36.6 ± 15.1%; p < 0.001) while remained stable in CSII group (40 ± 11% to 39.3 ± 10.3%). A1c decrease in 1.08% (p < 0.001), 0.64% (p < 0.001) and 0.38% (p = 0.01) at 6 months in relation to basal in the COMBO, GLIC and CSII respectively. Daily basal insulin dose was reduced by 8.8% (p = 0.01) in the COMBO group.

**Conclusion:**

The COMBO and a mobile applicative (GLIC) bolus calculator had a similar and a good performance to optimize the intensive insulin treatment of T1D in the public health system with increase in the TIR and reduction in A1C without increase hypoglycemia prevalence.

## Background

The Brazilian Type 1 Study Group (BrazDiab1SG), a multicenter study carried out in 20 public care centers reported that only 10% of Type 1 Diabetes (T1D) participants achieved glycated hemoglobin (A1c) within the target (< 7%), with the national A1c average in these patients being 9.1% [1]. Several factors related to poor glycemic control of these participants are educational level, economic status, age, duration of T1D, adherence to the dietary plan, number and frequency of hypoglycemia, difficult to access a specialized care, the inputs for daily minimum blood glucose measures, among other [2, 3]. Beside this, it had been shown that regardless of treatment insulin schedule 31% of Brazilian T1D people were also overweight, what might potentially increase of insulin resistance [4].

Optimizing postprandial glucose control is known to aid in the reduction of A1c, hypoglycemia risk, glycemic variability, and weight gain [5, 6]. However, A1c reductions are also markedly dependent to patient lifestyle, treatment adherence and self-attitude. Specifically, in T1D, where the endogenous insulin secretion is absent or very low, the good glycemic control depends on rapid insulin bolus. In this way, today, several tactics have been proposed to optimize these insulin boluses [7–10].

Omission of self-monitoring blood glucose levels and “forgetting” meals boluses correction, even by patients using continuous subcutaneous insulin infusion (CSII), are directly related to an increase in A1c [11]. Moreover, performing a complex insulin bolus calculation in according to blood glucose (BG) level, meal ingestion, past and present physical activities and target limits goals need to be considerate and could be collaborated to fail in this task. Also, fear of hypoglycemia is one of the main factors that hinders the issue of insulin bolus, partially because manual calculation increases its risk [12, 13].

By comparing manual and automated methods to calculate insulin boluses to cover all these factors in people with T1D, a study revealed an approximately tenfold higher error rate for manual than automated insulin boluses. Therefore, most individuals felt more confident (83%) and preferred (87%) to use the automated method for calculating insulin boluses [14].

Insulin bolus calculators, come together to CSIIs systems, however, it uses still restricted to a 7% of Brazilian T1D population, due to their high cost [2, 15].

Multiple daily insulin (MDI) injection therapy is the main form of intensive insulin therapy for people with Brazilin T1D, either with long insulin analogs associated with fast analogs, or more frequently yet with human NPH insulin associated with human short-acting insulin or fast analog. Beside they have insulin free of charge from by Brazilian Public Health System, many have low adherence to treatment, about self-monitoring of blood glucose (SMBG) and meal insulin bolus administration. Justifications to this behavior are many, as lack of inputs provided by public agencies, lack of time, irregular mealtimes, forgetting insulin bolus doses, or pain at the site of both the SMBG and the subcutaneous insulin infusion. A great percentage of MDI-treated T1D individuals use empirical insulin dose or worse, missed boluses. These became more complex and frustrating when we relate it to Brazilian socioeconomic conditions.

Hence, it is pertinent to explore the insulin bolus calculators, possible to use in a T1D population with poorly glycemic control assisted in a public health service to optimize MDI therapy.

## Methods

### Design

A prospective, controlled, randomized, parallel intervention trial for 24 weeks in a Brazilian Tertiary Public Health Center to compare three insulin bolus calculators to increase time in range (TIR) and decrease A1c in T1D adults with poor glycemic control.

All patients included in the study signed an informed consent form.

The inclusion criteria were age between 18 and 45 years old, T1D definition by The American Diabetes Association (ADA) criteria [16], on MDI (insulin basal/bolus) therapy with fast-acting insulin analogs or CSII without continuous glucose-sensor and A1c > 8.5%. The exclusion criteria were intestinal, liver, kidney diseases, psychopathy and hemoglobinopathies.

Initially 203 T1D adults were selected from the electronic medical record to participate in the study including CSII, GLIC® APP (Brazilian insulin bolus calculator application) and COMBO® (Smart Control Accu—Check COMBO® insulin bolus calculator).

The intervention groups studied were composed of 168 T1D patients, using MDI, randomized into COMBO (n = 82) and GLIC(n = 86) groups, there was a drop out of 49 people in the COMBO group and 43 people in the GLIC group.

**Fig. 1:**
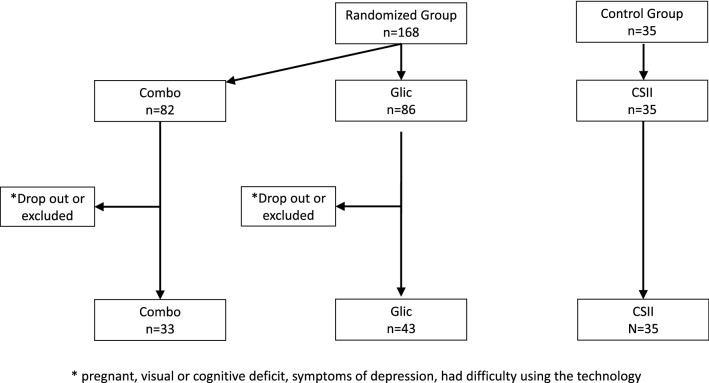
Recruitment and randomization flowchart of the studied groups

CSII group (n = 35): was the control group representing patients previously using CSII in the routine care of Diabetes Center (Fig. 1).

All participants underwent a period of diabetes education review and orientation (signs and symptoms of hyperglycemia, hypoglycemia and ketosis, guidance for sick days, exercise guidance, training on carbohydrate counting).

Total daily insulin dose (TD) was individualized through the calculation by patient’s weight multiplied by 0.7 to 1.0 U/Kg. Insulin Carbohydrate ratio and Sensitivity factor was calculated by the IR/CHO 450/TD, and FS = 1800/TD rule, and proper handling of technology implemented [17] was carried out by one of the investigators (VM).

### Insulin bolus calculators

GLIC^®^ APP: suggested insulin dose is based on an algorithm that considers the input of multiple variables, such as: sensitivity factors, carbohydrate-insulin ratio and fat-insulin, blood glucose value and targets, residual or active insulin, food intake (carbohydrates, fat, and other macronutrients).

COMBO^®^: bolus calculation considers some parameters: post-prandial glucose increasing, insulin duration, waiting time for decrease glucose, health events, and exercises, beyond the medical prescription (sensitivity factors, carbohydrate-insulin ratio, and glycemic target).

### Demographic, glycemic parameters and glycated hemoglobin evaluations

Anthropometric and clinical assessment were weight (kg), height(cm), waist circumference (WC) (cm), body mass index (BMI) (kg/m^2^) and blood pressure (mmHg).

Capillary blood glucose (mg/dL): the COMBO group used Perform^®^ reagent strips while the GLIC group used Gluco Leader^®^ Glucometer with Enhance II^®^ reagent strips, and data were extracted via the Glico Sys^®^ platform.

Time in Range (TIR) was percentage of time of capillary glycemic values between 70 and 180 mg/dL, extracted from glucometers upload.

Average blood glucose (AG): Blood glucose (mg/dL) was extracted from glucometer data uploaded the Accu-Chek Smart Pix^®^—Roche^®^ platform.

Glycemic targets for adjusting insulin doses in basal-bolus therapies were in according to ADA orientations, such as fasting and pre-prandial glycemia between 70 to 130 mg/dL and 2 h after meals < 180 mg/dL, respectively [18]. Patients were asked to attend diabetes clinic at Visit one (basal), Visit 2 (3 months) and Visit 3 (6 months) of follow-up. When necessary, phone calls completed the presential appointment. Fasting and post prandial glycemia was reassessed at each visit (Basal, 3 and 6 months).

Hypoglycemia: Hypoglycemia was considered capillary glycemia below 70 mg/dL.

Glycated Hemoglobin: high pressure chromatography, certified by the National Glycohemoglobin Standardization Program (NGSP) Reference Value: 3.4 to 5.6% [19].

### Diet, physical activity, and compliance evaluations

Diet adherence was defined as following at least 80% of the reported diet.

Physical activity: Participants were asked how many times physical activity was planned for each week and was classified as: Yes: physical activity performed 35 min three times a week, and No: less than three times a week or no activity reported.

Compliance with the intervention device was confirmed by the upload of insertion data of four capillary blood glucose per day and grams of carbohydrates into the GLIC app https://gliconline.net/entrar* and the Combo Accu-Chek Smart Pix*^*®*^*—Roche*^®20^.

Were considered adherence use of the intervention device during all the study time considering on uploaded data during appointment the detection of at least 3 capillary glucose measured by day.

### Statistical analysis

For descriptive analysis, continuous variables are expressed as summary measures (mean, median, standard deviation, and quartiles) while categorical variables are expressed as percentages.

To compare the groups in relation to continuous variables with repeated measures, nonparametric two-way ANOVA was used. For binary categorical variables, a mixed logistic model was used, with group as a fixed effect and individuals as a random effect.

To verify the difference between continuous measures within the same group, nonparametric one-way ANOVA was used, as well as the Mann–Whitney paired test for two-by-two comparisons.

For variables with single measures, the student’s t-test was used to compare groups in relation to continuous variables for variables that follow a normal distribution (Anderson–Darling test). The nonparametric Mann–Whitney and Brunner-Munzel tests were respectively used for homogeneous and heterogeneous variables (Bartlett test). For categorical variables, Fisher's exact test was used.

Linear regression was carried out withA1c variation as an outcome and Adherence and Group as explanatory variables. The significance level adopted in the tests was 0.05. Two-tailed hypotheses were considered. R software version 3.6.0 was used to perform all analyses.

## Results

### Enrollment and participants characteristics

The three groups studied had similar age range, diabetes duration and ketoacidosis frequency at diagnosis. Women frequency was greater in CSII group than in the intervention groups. These data are shown on Table 1.

**Table 1:** Baseline Characteristics of study group participants

	Mean ± SD or N (%)			P value		
	CSII	COMBO	GLIC	COMBO x CSII	GLIC X CSII	GLIC x COMBO
Continuous variables						
Age	27.82 ± 5.98	26.03 ± 7.04	26.81 ± 7.06	0.185^1^	0.343^1^	0.666^1^
Duration of diabetes	18.41 ± 6.54	16.47 ± 7.55	16.86 ± 6.07	0.173^1^	0.287^2^	0.512^1^
Categorical variables						
Gender (female)	26 (76.5%)	14 (43.8%)	24 (57.1%)	0.011^3^	0.093^3^	0.348^3^
Adherence (YES)	27 (79.4%)	24 (75.0%)	24 (63.2%)	0.772^3^	0.194^3^	0.314^3^
Ketoacidosis at diagnosis	15 (44.1%)	14 (43.8%)	22 (52.4%)	1.000^3^	0.498^3^	0.491^3^

*CSII:* Continuous Subcutaneous Insulin Infusion

1: Mann–Whitney; 2: t-Student test; 3: Exact Fisher Test

### Intra-groups Comparations

#### Clinical parameters and daily insulin dose

These measures shown differences only in COMBO group. In this group some clinical and daily insulin (IU/kg/day) parameters significantly different after 6 months of study. Basal insulin (IU/kg/day) was reduced by 8.8% (p = 0.01), weight increased 2% (p = 0.007), waist circumference (WC) increased 5.4% (p < 0.001) and systolic blood pressure (SBP) reduced in 8.1% (Table 2).

**Table 2:** Comparative clinical and glycemic variable intra groups studied at baseline, 3, and 6 intervention months

Variable	Baseline	Mean ± SD		Intervention period				
		3 months	6 months	0 × 3 months	3 × 6 months		0 × 6 months	
CSII group (n = 35)								
Basal	24.7 ± 8.41	24.97 ± 10.32	24.06 ± 22,90	–	–		0.731 (p2)	
SBP	114.18 ± 13.12	112,53 ± 12.01	112.85 ± 12.50	–	–		0.712 (p2)	
Weight	67.84 ± 10.6	68.19 ± 12.38	68.47 ± 11.67	–	–		0.523 (p2)	
A1c	9% ± 0.5%	8.6% ± 0.6%	8.6% ± 0.8%	0.005	0.858		0.010	
TIR	40.0% ± 11.3%	39.4% ± 10.3%	39.3% ± 10.5%	0.596	0.950		0.573	
AG	197.6 ± 28.1	197.1 ± 34.2	197.4 ± 30.4	0.917	0.963		0.963	
SD	91.2 ± 13.2	90.9 ± 17.4	91.0 ± 13.9	0.912	0.977		0.944	
Hipo < 70	6.5% ± 7.6%	7.1% ± 6.8%	5.8% ± 5.0%	0.402	0.369		0.902 (p2)	
Combo group (n = 33)								
Basal	28.03 ± 8.54	26.44 ± 8.69	25.56 ± 8.38	0.062	0.111		0.010	
SBP	123.59 ± 12.46	118.84 ± 11.25	113.56 ± 12.93	0.061	0.058		< 0.001	
Weight	70.46 ± 13.93	71.2 ± 14.39	71.83 ± 14.77	0.056	0.037		0.007	
WC	82.88 ± 11.13	85.69 ± 11.06	87.34 ± 11.67	< 0.001	0.017		< 0.001	
A1c	9.9% ± 1.3%	8.9% ± 1.2%	8.8% ± 1.3%	< 0.001	0.221		< 0.001	
TIR	28.9% ± 11.9%	40.5% ± 11.8%	38.3% ± 12.7%	< 0.001	0.193		< 0.001	
AG	224.51 ± 49.64	204.38 ± 38.98	209.75 ± 40.92	0.005	0.190		0.012	
SD	100.9 ± 28.1	96.8 ± 21.9	96.4 ± 20.6	0.395	0.906		0.191	
Hipo < 70	6.0% ± 7.2%	6.3% ± 4.8%	5.9% ± 3.9%	0.462	0.395		0.673 (p2)	
Glic group (n = 43)								
Basal	25.68 ± 8.14	25.13 ± 6.51	24.26 ± 7,07	–		–		0.229 (p2)
SBP	119.08 ± 1.34	113.82 ± 12.34	112.71 ± 9.10	–		–		0.125 (p2)
Weight	67.10 ± 11.68	67.03 ± 11.03	67.16 ± 10.94	–		–		0.911 (p2)
WC	82.81 ± 9.50	83.08 ± 9.07	83.89 ± 9.31	–		–		0.260 (p2)
A1c	9.6% ± 1.5%	9.2% ± 1.8%	9.0% ± 1.4%	0.001		0.065		< 0.001
TIR	27.9% ± 13.4%	33.9% ± 13.8%	36.3% ± 15.1%	0.003		0.200		< 0.001
AG	216.4 ± 38.89	205.32 ± 42.45	203.85 ± 44.99	0.016		0.810		0.039
SD	100.3 ± 23.24	92.17 ± 22.36	90.61 ± 24.16	0.002		0.648		0.033
Hipo < 70	5.6% ± 5.5%	6.1% ± 4.3%	6.6% ± 5.1%	0.426		0.939		0.437 (p2)

*CSII*^:^ Continuous Subcutaneous Insulin Infusion^,^ *A1c*^*:*^ Glycated Hemoglobin^,^ *Basal*^:^ Insulin basal rate^,^ *WC*^:^ Waist Circumference^,^ *SBP*^:^ Systolic Blood Pressure^,^ *TGT*^:^ Time on Glycemic Target^,^ *SD*^:^ Standard Deviation^,^ *AG*^:^ Average Glucose

#### Glycemic parameters

After 6 months of follow-up blood glucose mean reduced 5.8% in GLIC (216.4 ± 38.8 mg/dL (Mean ± SD) to 203.85 ± 44.99 mg/dL; p = 0.039), 7% in COMBO (224.5 ± 49.6 mg/dL to 209.7 ± 40.92 mg/dL (p = 0.012) and remained stable in CSII group (197.6 ± 28.1 mg/dL to 197.3 ± 30.4 mg/dL.

Blood glucose standard deviation (GSD) was reduced only in GLIC^®^ group (100.3 ± 23.2 mg/dL to 90.6 ± 24.1 mg/dL) at 6 months; p = 0.003) (Table 2).

**Fig. 2:**
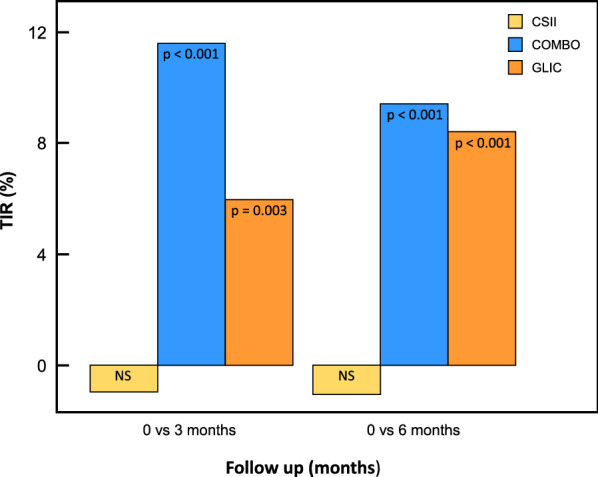
Comparative Analysis of Time in Range during follow up among the three groups

After 6 months of intervention TIR was increased in 9.42% in COMBO group (29 ± 12% to 38.9 ± 12.7%; p < 0.001) in 8.39% in the GLIC^®^ group (28 ± 15% to 36.6 ± 15.1%; p < 0.001) while remained stable in CSII group (40 ± 11% to 39.3 ± 10.3%) (Fig. 2).

**Fig. 3:**
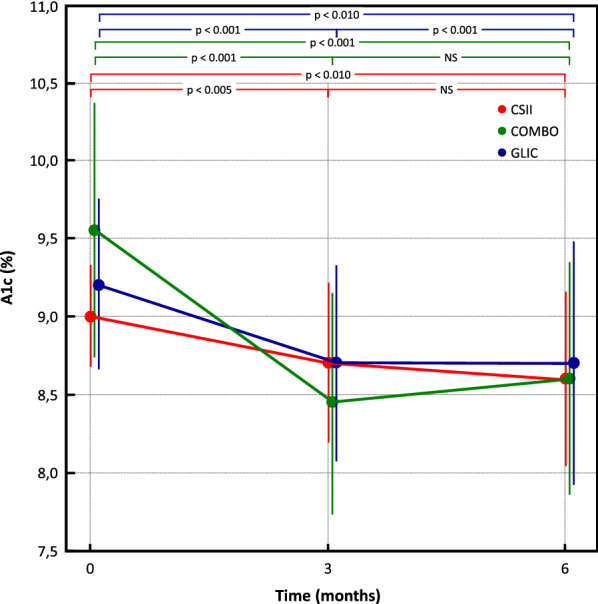
Graph showing a reduction in glycated hemoglobin in the three groups over the course of 6 months

A1c had a mean reduction of -1.08% (9.9% ± 1.3% to 8.8 ± 1.3%; p < 0.001) in COMBO^®^ Group, -0.64% in GLIC^®^ group (9.6 ± 1.5% to 9.0 ± 1.4%; p < 0.001), and -0.38% (9.0 ± 0.5% to 8.6 ± 0.8%; p = 0.01) in CSII group (Fig. 3).

### Inter group comparisons

#### Clinical parameters and daily insulin dose

WC was significantly different in GLIC^®^ and COMBO^®^ groups. In the baseline (82.81 ± 9.5 vs 82.88 ± 11.13 cm; p = 0.010) and after 6 months (83.89 ± 9.31 vs 87.34 ± 11.67; p = 0.010) respectively. While SBP was reduced only in the COMBO^®^ group, (123.59 ± 12.46 mmHg vs 113.56 ± 12.93 mmHg; p = 0.030) (Table 3).

**Table 3:** Clinical and Glycemic variable inter groups studied at baseline, 3, and 6 months

		Mean ± SD			p1		
		Baseline	3 months	6 months	Combo x CSII	Glic x CSII	Glic x Combo
IB	CSII	24.27 ± 8.41	24.97 ± 10.32	24.06 ± 7.68			
	COMBO	28.03 ± 8.54	26.44 ± 8.69	25.56 ± 8.38	0.022	0.362	0.251
	Glic	25.68 ± 8.14	25.13 ± 6.51	24.26 ± 7.07			
SBP	CSII	114.18 ± 13.12	112.53 ± 12.01	112.85 ± 12.5			
	COMBO	123.59 ± 12.46	118.84 ± 11.25	113.56 ± 12.93	0.030	0.722	0.322
	Glic	119.08 ± 15.34	113.82 ± 12.34	112.71 ± 9.1			
TIR	CSII	40.4% ± 11.3%	39.4% ± 10.3%	39.3% ± 10.5%			
	COMBO	28.9% ± 11.9%	40.5% ± 11.8%	38.3% ± 12.7%	< 0.001	0.002	0.116
	Glic	27.9% ± 13.4%	33.9% ± 13.8%	36.3% ± 15.1%			
AG	CSII	197.65 ± 28.1	197.12 ± 34.23	197.38 ± 30.43			
	COMBO	224.51 ± 49.64	204.38 ± 38.98	209.75 ± 40.92	0.064	0.277	0.435
	Glic	216.4 ± 38.89	205.32 ± 42.45	203.85 ± 44.99			
Bolus	CSII	26.15 ± 11.89	29.48 ± 11.31	28.03 ± 9.63			
	COMBO	26.16 ± 10.34	26.31 ± 11.79	29.69 ± 12.66	0.354	0.029	0.289
	Glic	28.47 ± 11.58	24.29 ± 9.84	27.53 ± 11.47			
WC	COMBO	82.88 ± 11.13	85.69 ± 11.06	87.34 ± 11.67			0.011
	Glic	82.81 ± 9.5	83.08 ± 9.07	83.89 ± 9.31			
A1c	CSII	9.0% ± 0.5%	8.6% ± 0.6%	8.6% ± 0.8%			
	COMBO	9.9% ± 1.3%	8.9% ± 1.2%	8.8% ± 1.3%	0.053	0.931	0.050
	Glic	9.6% ± 1.5%	9.2% ± 1.8%	9.0% ± 1.4%			

*CSII*^:^ Continuous Subcutaneous Insulin Infusion^,^ *A1c*^*:*^ Glycated Hemoglobin^,^ *Basal*^:^ Insulin basal rate^,^ *WC*^:^ Waist Circumference^,^ *SBP*^:^ Systolic Blood Pressure^,^ *TGT*^:^ Time on Glycemic Target^,^ *SD*^:^ Standard Deviation^,^ *AG*^:^ Average Glucose

p1 = refers to nonparametric ANOVA with two way about the comparison between baseline time and 6 months between groups

Total basal insulin administration in COMBO^®^ was higher than CSII Group at time zero (28.0 ± 8.5 vs 24.27 ± 8.41 IU/day; p = 0.022) (Table 3).

The total dose of bolus at 6 months follow up at GLIC^®^ was significantly lower than CSII group (27.53 ± 11.47 vs 28.3 ± 9.63 IU/Kg/day; p = 0.029) (Table 3).

#### Glycemic parameters

Blood glucose mean shown a tendence (p = 0.06) to significance difference only between COMBO vs CSII (209.75 ± 40.92 vs 197.38 ± 30.43 mg/dL) groups after 6 months intervention (Table 3).

TIR was significant different between COMBO group and CSII group 38.3% 12.7% ± vs 39.3% ± 10.5%; p < 0.001 and GLIC group vs CSII group (33.3% ± 15.1% vs 39.3% ± 10.5%; p = 0.002) at 6 months of follow-up (Table 3).

A1c was significantly different between COMBO vs CSII groups (8.8% ± 1.3% vs 8.6% ± 0.8%; p = 0.05) and COMBO vs GLIC groups (8.8% ± 1.3% vs 9.0% ± 1.4%; p = 0.05) at 6 months of follow-up (Fig. 3).

There was no significant difference in hypoglycemia frequency among all groups at any time of the study: basal, 3 and 6 months (Table 2).

## Discussion

The use of two different technologies (GLIC^®^) and Smart control glucometer (COMBO^®^) as a bolus calculator (> 3 capillary blood glucose measures a day) significantly increased TIR and reduced A1c without increasing hypoglycemia prevalence in a group of adults T1D with poor glycemic control from a Public Diabetes Care Center.

After 6 months of intervention during follow-up TIR increased in 9.42% in COMBO^®^ group and 8.39% increase in the GLIC^®^ group. At the end of the study (6 months of follow-up) TIR of GLIC^®^ (36.3%), COMBO^®^ (38.3%) and CSII (40%) groups were similar. Beside of these TIRs still far from the minimum goal of TIR propose to a good glycemic control (> 70%) it was like TIR found in the DCCT participants were also using SMBG (41 ± 16%). The increase in TIR got in the COMBO^®^ and GLIC^®^ groups (9.4 and 8.4% respectively) was near to 10% that was shown to be sufficient to reduce the risk to diabetic retinopathy and microalbuminuria [21]. Recently these data was confirmed by the International Consensus for Continuous Monitoring where was demonstrated that 5% increase in TIR contributes to reduce chronic diabetic complications, so an increase of almost 9% in TIR in our intervention groups has potentially a clinical impact to reduce diabetes complications [22]. We also found a decrease in mean blood glucose in the GLIC^®^ and COMBO^®^ groups as in blood glucose SD in the GLIC^®^ group, two important components of glycemic variability.

Glycemic variability (GV), referring to oscillations in blood glucose levels, is usually defined by the measurement of fluctuations of glucose or other related parameters of glucose homeostasis over a given interval of time (i.e., within a day, between days or long term). GV is considered independent risk factors for diabetes-related complications related to possible vascular damage due to excessive glucose fluctuations and an increased risk of hypoglycaemia, and can be better evaluated using Mean Glycemia, standard deviation and its coefficient of variation (CV = AG/SD × 100). [23]

Both Daily or Between Days short-term or long-term glycemic variability (A1c) should be considered independent risk factors for diabetes-related complications like an increased risk of a major cardiovascular diseases, diabetic retinopathy, sensory neuropathy, diabetic nephropathy, predisposes symptoms of depression and association with decline in cognitive function. It has also been associated with an increased risk of mortality in diabetic population. [24]

The reduction of A1c was more in COMBO^®^ group (1.08%) than in GLIC^®^ group (0.64%). This could be a result of less adherence to the GLIC^®^ applied, probably due to various steps to reach final insulin bolus calculation. Nevertheless, a meta-analysis that evaluated the effectiveness of cell phone application in diabetes management and storage and collection of data for healthcare team analysis revealed a 0.44% decrease in A1c [25]. This systematic review and meta-analysis of efficacy of mobile APPs to support care of people with diabetes, confirmed that A1c could be improved when it strengthens the perception of self-care and allow remote access to health care professional, but an important characteristic measured in these studies was participants education degree and the ability in adopting new technologies [25]. In addition, a recent Brazilian study carried out with a group of adults with T1D or latent autoimmune diabetes of adult (LADA), undergoing treatment with MDI plus carbohydrate counts or MDI plus fixed doses of insulin compared to CSII also observed that using SMBG device connected to a cell phone application contributed to better glycemic control and increased adherence to tasks of self-care [26].

Specifically in relation to A1c reduction (1.08%) in COMBO^®^ group those might reflect better insulin treatment adherence with this device. This result on A1c was better than it was found by the ABACUS study (reduction of 0.7% in A1c) when evaluating T1D that used the Accu-Check Aviva Expert BG meter^®^ bolus calculator that resembles the COMBO^®^ device use in our study [12].

Comparation between CSII and GLIC^®^ groups shown that there was insulin dose bolus reduction in the GLIC^®^ group (3.3%) while there was an increase of 7.1% in the CSII group. This reflects better adherence to bolus in the CSII group due to easiness of self-explanatory management of the device determining the exact basal and bolus dose in the profile while GLIC^®^ device depends on data entry, which is often inputted irregularly. However, there was an increase in TIR and one reduction in A1c, as there was an increase in adherence to meal insulin bolus due to ease of carbohydrate counting by APP.

The reduction in basal/bolus insulin dose ratio in COMBO^®^ group can reflect an optimization of insulin therapy in this group resulting greater A1c reduction. Optimized metabolic control was also demonstrated by WC increased, SBP reduction without raising in hypoglycemia prevalence.

Although cost-effective analyses were not done in this study, it is important to consider that the Smart Control Accu-Check COMBO^®^ Bolus Calculator costs (~ US$ 243.00) and 150 reagent strips/month (~ US$ 50.00) but it was free of charge from SUS) to use the GLIC^®^ are cheaper than CSII costs (~ US$ 2,727.00 per unit plus US$ 181.00 a month by supplies). [27, 28]

In our study, all groups used SMBG but is important to comment that better results would have been possible if continuous glucose monitoring system (CGMS) were used, as demonstrated by COMISAIR study were CGMS impacted glycemic outcomes more than insulin delivery method, CSII or MDI [29].

### Study limitations

Herein, we performed a prospective study using low-income T1D people from the Public Health System. Of note, there was a high frequency of participants drop out due to socioeconomic conditions, such as difficulty in maintaining internet or cell phone data. Another limitation was we did not evaluate our participant’s education level because results favoring intervention groups might have been better if they had more scholar education degree.

The GLIC^®^ bolus calculators were performed after the insertion of blood glucose measured by a glucometer provided by the SUS but sometimes, they were measured but not inserted in the APP. In addition, both COMBO device bolus calculator and the GLIC^®^ used SMBG instead of CGM, which made it difficult to assess hypoglycemia. However, it reflects real-life challenge faced by most of T1D Brazilian population who do not have access to CGM [30, 31].

People with T1D still struggle to attain ideal glycemic control and avoid long-term complications despite these advancements in diabetes technology and diabetes education. The major cause of this can be attributed to the huge number of variables that influence the glucose control and must be considered when determining how much insulin is required for a meal. Some factors were not studied in this paper as low education level, smoking, living alone, exercising infrequently, nutrition factors which include low intake of fruit and vegetables, use of caffeine, alcohol consumption, biological or pathologic factors as menstruation, and celiac disease [32].

Finally, it is worth considered that this study was done during Covid-19 pandemic which made difficult to patients attend all presential appointment.

## Conclusion

This study demonstrated that administration of intensive insulin treatment to poor glycemic control (A1c > 8.5%) T1D adults from public health system, can be optimized (reduction in A1c and an increase in TIR without an increase in hypoglycemia), using bolus calculators by intelligent glucometer (COMBO^®^) or mobile APP (GLIC^®^) became its like CSII users.

Thus, the facilitators in basal/bolus insulin therapy associated to seek guidance from professionals trained in diabetes education should be used to overcome personal and general difficulties found in a health system of developing countries to achieve better glycemic control and potentially collaborate to reduce the precocious diabetes chronic complications frequently found in these patients.

## Data Availability

The datasets used and/or analyzed during the current study are available from the corresponding author on reasonable request.
